# Can Molecular Attributes of Mammalian Granulosa Cells and Ovarian Putative Stem Cells Predestine Them to Be a Promising Tool for Tissue Engineering and Regenerative Medicine?

**DOI:** 10.3390/ijms262110667

**Published:** 2025-11-01

**Authors:** Małgorzata Duda, Marcin Samiec

**Affiliations:** 1 Department of Endocrinology, Institute of Zoology and Biomedical Research, Faculty of Biology, Jagiellonian University in Krakow, Gronostajowa 9 Street, 30-387 Krakow, Poland; maja.duda@uj.edu.pl; 2 Department of Poultry Breeding, National Research Institute of Animal Production, Krakowska 1 Street, 32-083 Balice, Poland

**Keywords:** granulosa cells, ovarian putative stem cells, multipotency, pluripotency-related transcription factors, molecular plasticity, neurogenic and endotheliogenic transdifferentiation, epigenetic modulation, DNA methyltransferase inhibitor, reconstructive medicine, gynecological oncology

## Abstract

Granulosa cells (GCs) and ovarian putative stem cells (oPSCs) represent distinct but complementary populations within the mammalian ovary. While GCs have long been considered terminally differentiated and hormonally specialized, emerging evidence indicates that they retain epigenetic plasticity and, under defined conditions, can be reprogrammed into cells exhibiting pluripotent-like features. In contrast, oPSCs, including oogonial stem cells (OSCs) and very small embryonic-like stem cells (VSELs), are naturally multipotent and capable of spontaneous or inducible differentiation into neural, endothelial, and other somatic lineages. Both cell types express stemness-related markers, such as OCT4, SOX2, and c-KIT, and demonstrate potential for self-renewal and lineage conversion. Recent advances in chemical modulation of epigenetic reprogramming, particularly with agents from the family of non-specific DNA methyltransferase (DNMT) inhibitors, such as 5-azacytidine (5-azaC), highlight the feasibility of generating functional, lineage-specific derivatives of GCs or oPSCs without genetic manipulation. Not without significance is also the fact that extended/high-dose 5-azaC-mediated modulation can induce cell senescence or apoptotic/necrotic death. Therefore, dosing must be carefully titrated, which strongly supports a dose- and/or time-dependent mechanism for 5-azaC-based epigenetic modification in treated cells. This study aims to summarize the molecular and functional properties of mammalian GCs and oPSCs, emphasizing their applicability in regenerative medicine and reproductive bioengineering, with a focus on safe, patient-specific cell-based therapies.

## 1. Introduction

**Figure 1:**
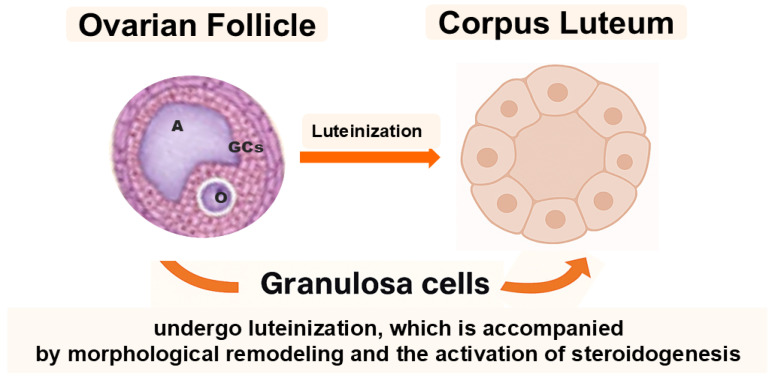
Granulosa cells (GCs) surround the oocyte (O) and are subdivided into cumulus and mural granulosa cells. The antrum (A) develops within the follicle during maturation. After ovulation, mural GCs undergo luteinization to form the corpus luteum, accompanied by profound morphological and steroidogenic changes.

The mammalian ovary is not only the site of oogenesis and steroidogenesis but also a dynamic organ containing cell subpopulations with remarkable plasticity. Among these, granulosa cells (GCs) and ovarian putative stem cells (oPSCs) have emerged as key cellular elements that transcend their classical reproductive functions and may offer promising avenues for regenerative medicine. Historically considered terminally differentiated or lineage-restricted, both GCs and oPSCs have been shown to exhibit surprisingly broad cellular plasticity, particularly under specific in vitro culture conditions or upon epigenetic modulation [1,2,3,4]. Reports on human oPSCs remain contested; standardized isolation, lineage tracing, and safety validation are needed before translation. In the ovarian follicle, GCs play a fundamental role in supporting oocyte growth and follicular development. They are typically subdivided into cumulus cells, which directly surround and nourish the oocyte, and granulosa cells, mural and antral, that line the follicular wall and contribute to steroidogenesis. Following ovulation, GCs undergo luteinization, a process that leads to the formation of the corpus luteum and is accompanied by profound morphological remodeling and enhanced steroid hormone production (Figure 1) [5].

Emerging studies have demonstrated that mammalian GCs can acquire stem-like properties, including self-renewal capacity, expression of pluripotency-associated transcription factors (e.g., OCT4, SOX2, and NANOG), and the potential to transdifferentiate into mesodermal, endodermal, or ectodermal derivatives [6]. In turn, oPSCs, including their crucial representatives such as very small embryonic-like stem cells (VSELs) and oogonial stem cells (OSCs), have been found to exhibit multipotency-related attributes under specific conditions. For this reason, their true nature remains debated. The ovarian PSCs reside in the ovarian cortex, suggesting they may participate in postnatal folliculogenesis or contribute to tissue repair. Therefore, the identification of rare subpopulations of adult ovarian stem-like cells, capable of clonal expansion and oocyte- or somatic cell-like differentiation, has challenged the long-held belief in the finite ovarian reserve and not only opened up novel avenues for reproductive biology, regenerative medicine and personalized reconstructive therapies arising from gynecological oncology-related surgical approaches, but also provided new mechanistic insights into cellular reprogramming [7,8,9].

The molecular plasticity of GCs and oPSCs appears to be underpinned by complex mechanisms, notably epigenetic regulation. Epigenetic modifications such as DNA methylation, histone tail modifications, and non-coding RNAs play fundamental roles in defining and maintaining cell identity [10,11,12]. They also provide a reversible and tunable layer of regulation that can be exploited to reprogram cell fate without altering the genomic sequence. Recent progress in epigenetic editing, stem cell engineering, bioinformatics, and single-cell multimodal omics profiling or sequencing has reinforced the idea that GCs and oPSCs may represent ethically acceptable, autologous sources for personalized regenerative therapies [13,14].

This study aims to provide a comprehensive synthesis of current knowledge on the molecular attributes of GCs and oPSCs, with particular emphasis on their regenerative potential. This study also seeks to comparatively explore not only the developmental origins of GCs and oPSCs, but also their molecular signatures, epigenetic landscapes, and capacities for reprogramming and differentiation. Furthermore, this study focuses on a meticulous examination of how these properties can be harnessed in tissue engineering, emphasizing key breakthroughs, current challenges, and future directions. Particular attention is given to the translational relevance of these findings in reproductive medicine, regenerative therapies, and disease modeling.

## 2. Granulosa Cells: More than Just Endocrine Cells

### 2.1. Cellular and Functional Identity

The mammalian ovary is a complex organ that supports not only oogenesis and steroidogenesis but also harbors cell populations exhibiting significant plasticity. Among these, granulosa cells (GCs) have attracted particular attention for their regenerative and stem-like potential extending beyond classical endocrine functions. Traditionally regarded as terminally differentiated and hormonally specialized somatic cells, GCs have been shown to reacquire stem-like properties under specific in vitro conditions, largely through epigenetic reprogramming [15,16,17].

Recent in vitro and ex vivo studies have demonstrated that GCs can express pluripotency-associated transcription factors such as OCT4, SOX2, and NANOG, exhibit self-renewal, and differentiate into cell types representing all three germ layers [18]. Understanding the molecular and functional identity of GCs—including their developmental provenance, epigenetic landscape, and capacity for plasticity and lineage-specific differentiation—offers valuable insights into how these features can be harnessed in tissue engineering and regenerative medicine, particularly within gynecological oncology.

### 2.2. Molecular Signature and Stemness-Related Markers

Although traditionally viewed as a terminally differentiated subpopulation, GCs display an unexpectedly high expression profile of stemness-associated genes and proteins. Numerous studies have confirmed the presence of OCT4 (POU5F1), SOX2, NANOG, and c-KIT (CD117) in GCs under various physiological and experimental conditions [12,13]. These markers—typically linked to embryonic stem cells (ESCs) and induced pluripotent stem cells (iPSCs)—suggest that GCs may retain or reactivate a latent developmental program.

Expression levels vary with the developmental stage of the ovarian follicle and the species studied. For instance, in bovine GCs, OCT4 and SOX2 are expressed more strongly in cells derived from small preantral follicles than in those from larger, more differentiated follicles [13]. Similarly, porcine and human GCs can maintain expression of pluripotency markers during long-term in vitro culture, particularly under conditions enriched with leukemia inhibitory factor (LIF) and basic fibroblast growth factor (bFGF) [16,17].

Microenvironmental conditions critically affect stemness maintenance. Three-dimensional (3D) spheroid culture and low-attachment systems promote cytoskeletal reorganization and STAT3 activation, a downstream signal of the LIF receptor that upregulates OCT4 and SOX2 [18,19]. Concurrent activation of PI3K/AKT and MAPK/ERK pathways has also been observed, supporting the persistence of stem-like features in GCs [20].

Additional stem cell markers, such as SSEA4, TRA-1-81, and CD133, have been identified in luteinized human GCs collected during IVF procedures. Furthermore, treatment with the DNA methyltransferase inhibitor 5-azacytidine (5-azaC) increases OCT4 and NANOG expression, leading to colony formation with alkaline phosphatase activity and pluripotent-like morphology [21,22,23].

Species-specific differences further modulate these properties. For instance, NANOG expression in porcine GCs exposed to LIF was more than twofold higher compared with untreated controls, highlighting the interplay between extrinsic cues and intrinsic epigenetic programming. Beyond marker expression, GC plasticity also reflects their metabolic profile. Cells with high OCT4 and NANOG levels show enhanced oxidative phosphorylation gene expression, characteristic of proliferating and reprogrammed cells, suggesting a close coupling between stemness and bioenergetic reprogramming [24,25,26,27].

Another important feature of GCs is their clonogenic potential. Under 3D culture conditions with LIF supplementation, both human and porcine GCs can form sphere-like colonies that retain high expression of stemness markers [28]. Notably, lentiviral reprogramming using only OCT4 and SOX2—without c-MYC or KLF4—has successfully generated iPSC-like colonies from human GCs, underscoring their intrinsic reprogrammability and clinical relevance [25,26].

Single-cell RNA sequencing (scRNA-seq) analyses further support the molecular heterogeneity of GCs during folliculogenesis. Although fully pluripotent subpopulations have not been identified, dynamic, stage-specific expression of developmental and regenerative genes is observed across mural and cumulus GC subsets [27]. This transcriptional diversity suggests that a fraction of GCs remains in a facultative progenitor-like state, capable of activating regenerative programs in response to defined stimuli.

Taken together, GCs possess a molecular and functional toolkit enabling stem cell-like behavior under appropriate conditions, positioning them as attractive candidates for patient-specific, ethically acceptable regenerative applications.

### 2.3. Molecular Plasticity, Epigenetic Reprogrammability, and Transdifferentiation Potential

**Figure 2:**
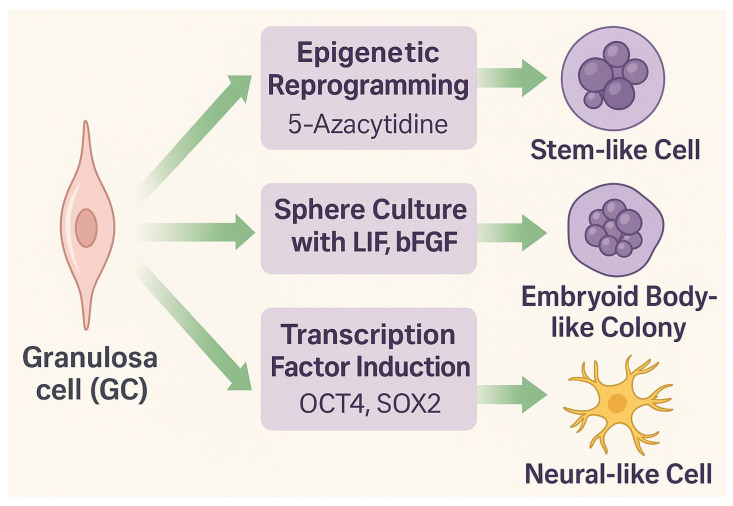
Experimental approaches to propagate stem-like properties in granulosa cells (GCs). Epigenetic reprogramming triggered by exposure to 5-azacytidine, sphere culture in the presence of LIF and bFGF, or chemically assisted induction mediated by a defined cocktail of transcription factors (OCT4, SOX2) can promote stemness, leading to the generation of embryoid body-like colonies or neural-like cells.

Granulosa cells represent a somatic cell population with remarkable epigenetic flexibility and the ability to adopt stem-like or lineage-specific phenotypes. Although originating from the coelomic epithelium and serving primarily reproductive and endocrine functions, GCs can be reprogrammed or transdifferentiated under controlled conditions. Experimental strategies to induce stemness include exposure to DNA methyltransferase (DNMT) inhibitors such as 5-azacytidine (5-azaC), sphere culture in the presence of growth factors (LIF, bFGF), or forced expression of transcription factors (OCT4, SOX2) (Figure 2) [17,28].

A notable approach involves epigenetic modulation with 5-azacytidine, which induces demethylation and reactivation of pluripotency genes (*OCT4*, *NANOG*, *SOX2*) [17,29]. The treated cells acquire a rounded morphology, alkaline phosphatase activity, and other features resembling those of embryonic stem cells (ESCs). However, prolonged or high exposure may lead to senescence or apoptosis, underscoring the need for precise dose and timing optimization.

GCs cultured under low-attachment or 3D conditions enriched with LIF and bFGF form sphere-like colonies, maintaining elevated stemness gene expression and self-renewal potential. These systems bypass contact inhibition and activate STAT3, PI3K/Akt, and Wnt/β-catenin signaling pathways, all of which contribute to enhanced developmental signaling.

Giorgetti et al. [16] demonstrated that human GCs can form iPSC-like colonies using only OCT4 and SOX2, avoiding oncogenic factors such as c-MYC and KLF4. These colonies exhibited ESC-like morphology, expression of pluripotency markers, and trilineage differentiation potential, confirming GCs’ suitability for autologous regenerative applications.

Moreover, reprogrammed GCs can be directed toward specific somatic lineages. Under neural induction, they upregulate βIII-tubulin (TUBB3), Nestin, and GFAP, acquiring neuronal morphology [30]. Comparable protocols induced osteogenic and chondrogenic differentiation, confirmed by mineralization assays and SOX9 expression [31]. These findings highlight GCs’ potential for transdifferentiation and their translational value for tissue-specific therapies.

Importantly, GCs’ transcriptional flexibility may stem from residual developmental memory. Despite differentiation, they maintain open chromatin regions near early embryonic regulatory loci, enabling rapid activation of stemness-related genes [32]. ATAC-seq studies confirm increased chromatin accessibility upstream of pluripotency genes, indicating a poised epigenetic state [33].

In summary, granulosa cells can be epigenetically and functionally reprogrammed into stem-like or lineage-specific phenotypes under defined conditions. Their availability, ethical acceptability, and responsiveness to reprogramming protocols make them promising candidates for regenerative medicine, tissue engineering, and disease modeling.

## 3. Ovarian Putative Stem Cells (oPSCs)

### 3.1. Isolation and Phenotypic Features

Ovarian putative stem cells (oPSCs), also referred to as ovarian stem cells (OSCs), have been identified in the ovarian cortex of several mammalian species, including humans, mice, and pigs. However, DDX4/SSEA-4-based isolation remains controversial and requires standardized validation. The discovery of these cells has challenged the long-held belief in the finite nature of the mammalian ovarian reserve and highlighted the regenerative potential of the adult ovary [34].

Ovarian PSCs are primarily localized within the tunica albuginea and cortical stromal regions, forming a heterogeneous population that can be enriched by fluorescence-activated cell sorting (FACS) or magnetic-activated cell sorting (MACS). The most commonly used antibodies target SSEA-4, CD34, IFITM3 (FRAGILIS), and DDX4 (VASA) [35,36]. In addition to their localization within the ovarian cortex and tunica albuginea, oPSCs have also been reported in the ovarian surface epithelium (OSE), suggesting the presence of multiple stem cell niches within the ovary [1,2]. This approach enriches a population of small cells (4–10 µm), which are often lost during standard centrifugation unless higher centrifugal forces (~1000× *g*) are applied.

Phenotypically, oPSCs co-express pluripotency markers such as OCT4, SOX2, NANOG, and DPPA3 (STELLA), as well as germline markers including VASA, DAZL, and c-KIT. This dual expression pattern suggests a hybrid germline–mesenchymal identity [37]. Additionally, oPSCs express mesenchymal stem cell (MSC)-related markers—CD90, CD105, CD73, and CD29—supporting their plasticity and multilineage potential [38]

Two major oPSC subpopulations have been described:

- Very small embryonic-like stem cells (VSELs): <5 µm; SSEA-4^+^/OCT4A^+^/CD34^+^/CD45^−^; typically quiescent with nuclear OCT4 expression [36].
- Oogonial stem cells (OSCs): 8–10 µm; DDX4^+^ with cytoplasmic OCT4 localization and higher mitotic activity compared with VSELs [37].

These subtypes likely represent different stages of developmental plasticity or lineage commitment. Under defined conditions supplemented with EGF, bFGF, and LIF, oPSCs can be expanded in vitro, maintaining survival, proliferation, and stem-like features [39].

Species-specific traits have been noted. In humans, DDX4^+^/SSEA-4^+^ cells are isolated from the ovarian cortex and form oocyte-like structures in vitro, though these findings remain controversial due to concerns about marker specificity. Conversely, porcine oPSCs (poPSCs) isolated from prepubertal ovarian tissue display MSC-like morphology and differentiate into endothelial-like cells under VEGF stimulation [38].

Epigenetic profiling has shown open chromatin and hypomethylated promoters of pluripotency genes (e.g., *OCT4*, *NANOG*), supporting their transcriptional accessibility and functional plasticity [34,37].

In summary, oPSCs represent a rare but potentially pluripotent ovarian cell population with a combined germline and mesenchymal molecular signature. Their in vitro behavior demonstrates functional plasticity that can be exploited in reproductive biology, regenerative medicine, and tissue engineering.

### 3.2. Differentiation Capacity

**Figure 3:**
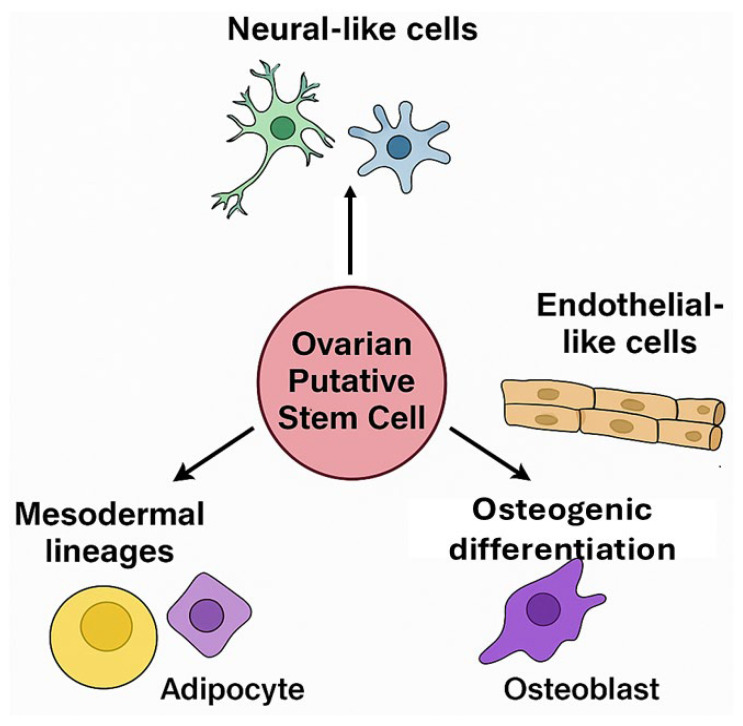
Differentiation potential of ovarian putative stem cells (oPSCs). oPSCs isolated from the ovarian cortex have been found to differentiate into diverse cell lineages, including neural-like cells, endothelial-like cells, adipocytes, and osteoblasts, highlighting their multipotency and responsiveness to niche-specific cues.

Ovarian PSCs from human and porcine ovaries exhibit broad differentiation potential in vitro, confirming their classification as multipotent stem cells. Their ability to acquire distinct phenotypes in response to specific cues underscores their translational value for tissue engineering and disease modeling (Figure 3).

Under neuroinductive conditions, porcine oPSCs exposed to forskolin and retinoic acid undergo neural-like differentiation. These treatments induce morphological changes (elongated bipolar or multipolar shapes) and upregulate neural markers, including NeuN, NPY, Nestin, and GFAP, as confirmed by immunocytochemistry and RT-PCR [40,41]. Such neurogenic plasticity supports their potential for neural repair and for modeling neurodegenerative diseases.

Beyond neurogenesis, oPSCs display vasculogenic and angiogenic capacity. When cultured in VEGF-enriched media, oPSCs differentiate into angioblast-like and endothelial-like cells, forming capillary-like networks and expressing vascular markers (VE-cadherin/CD144, PECAM-1/CD31, VEGFR2, and VEGFR3) [42]. These findings demonstrate their ability to contribute to neovascularization, supporting applications in vascular grafting and engineered tissue perfusion.

In addition, oPSCs differentiate into mesodermal lineages such as adipocytes and osteoblasts. In adipogenic conditions, they accumulate lipid droplets and express PPARγ and FABP4, while osteogenic induction triggers calcium deposition and RUNX2 and osteocalcin expression [43,44]. These results are consistent with their MSC-like phenotype (CD90^+^, CD105^+^, CD73^+^).

Emerging data indicate limited endodermal differentiation potential. Protocols using activin A, HGF, and nicotinamide have induced hepatocyte-like and pancreatic-like phenotypes expressing ALB, CYP3A4, and insulin [45].

Differentiation efficiency strongly depends on the extracellular matrix and mechanical environment. Culture substrate (e.g., Matrigel, collagen I), growth factor combinations, and hypoxia significantly modulate lineage outcomes. At the same time, chromatin accessibility at lineage-specific promoters facilitates responsiveness to external signals, as shown in ChIP-seq and ATAC-seq studies [45,46].

Collectively, oPSCs display neurogenic, vasculogenic, mesodermal, and potentially endodermal plasticity, underscoring their versatility and regenerative potential. Future research should validate their lineage stability, long-term functional integration, and safety before clinical translation.

### 3.3. Functional Integration and Electrophysiological Properties

The acquisition of functional electrophysiological activity is a key criterion confirming neural differentiation of oPSCs. Studies demonstrate that oPSC-derived neural-like cells exhibit resting membrane potentials, voltage-gated ion currents, and synaptic signaling competence—hallmarks of neuronal functionality.

Whole-cell patch-clamp recordings revealed delayed rectifier potassium currents and transient sodium currents, indicating membrane excitability and the integration of ion channels essential for action potential propagation and synaptic transmission [47,48].

In co-culture with rat hippocampal neurons, oPSC-derived neural-like cells exhibit morphological plasticity and intercellular connectivity. Within 7–14 days, they extend neurite-like projections, align with axonal tracts, and establish synaptic contacts, as evidenced by synapsin-1, PSD-95, and NR1 immunostaining [49].

Electrophysiological recordings during co-culture reveal spontaneous postsynaptic currents (sPSCs), confirming the establishment of functional synaptic input. Although their excitability remains immature compared to adult neurons, these cells can integrate into neural networks and transmit electrical signals, demonstrating neurophysiological competence.

Calcium imaging further supports neuronal functionality. Upon stimulation with KCl, glutamate, or ATP, differentiated oPSCs exhibit robust intracellular Ca^2+^ transients, visualized with Fluo-4 AM, reflecting activation of ionotropic glutamate receptors (AMPA/NMDA) and voltage-gated calcium channels (VGCCs) [50,51].

Transcriptomic profiling corroborates functional maturation, showing upregulation of ion channel genes (*KCNQ2*, *SCN1A*, *CACNA1C*) and synaptic scaffolding components. While full in vivo integration requires validation in preclinical models, current data strongly indicate that oPSC-derived neurons are functionally mature. Their accessibility, autologous origin, and electrophysiological activity position oPSCs as promising candidates for regenerative neurology, including applications in stroke recovery, Parkinson’s disease, and neurotoxicity testing [52].

## 4. Epigenetic Reprogramming of Granulosa Cells

### 4.1. Mechanisms of Epigenetic Plasticity

Epigenetic reprogramming enables terminally differentiated granulosa cells (GCs) to regain pluripotency features or transdifferentiate into other lineages. This process involves reversible chromatin modifications—DNA methylation and demethylation, histone remodeling, and microRNA (miRNA)-mediated regulation—without altering the DNA sequence.

One of the best-characterized tools for GC reprogramming is 5-azacytidine (5-azaC), a cytidine analog and non-selective inhibitor of DNA methyltransferases (DNMTs). When incorporated into DNA, 5-azaC induces global hypomethylation, particularly in CpG-rich promoters, leading to the re-expression of pluripotency genes such as *OCT4*, *NANOG*, and *SOX2*. These molecular changes are accompanied by a rounded, undifferentiated morphology and increased expression of mesenchymal markers (CD105, CD90), suggesting a shift toward a multipotent state [17,53,54,55].

Histone modifications further regulate chromatin accessibility. Acetylation of histone residues (H3K9, H4K8) by histone acetyltransferases (HATs) promotes open chromatin and transcription, whereas deacetylation by HDACs reverses this state. During GC reprogramming, enhanced histone acetylation at pluripotency gene promoters correlates with their activation, while repressive marks (H3K27me3) decline, weakening the differentiated cell identity [55,56].

MicroRNAs act as post-transcriptional modulators of stemness. miR-145 suppresses OCT4 and SOX2, and its downregulation during GC reprogramming lifts this inhibition, enabling pluripotency gene expression [57]. Other miRNAs (e.g., miR-21, miR-125b, miR-302, and miR-372) promote cell survival and block differentiation or apoptosis [57,58].

Genome-wide ATAC-seq analyses have revealed that GCs maintain open chromatin regions near homeotic and pluripotency gene loci, indicating a partially accessible epigenetic landscape even in differentiated cells. This “epigenetic memory” may derive from their cyclic endocrine activity and dynamic role in folliculogenesis [55,56,59].

In summary, GC plasticity results from coordinated DNA demethylation, histone modifications, and miRNA regulation, which collectively enable cells to escape a fixed differentiated phenotype and reacquire stem-like or lineage-specific traits. Understanding these mechanisms provides a foundation for developing safe, efficient reprogramming strategies for regenerative and reproductive medicine.

### 4.2. Experimental Approaches

Chemical reprogramming of porcine GCs offers a transgene-free, low-risk strategy for inducing cellular plasticity. Unlike viral methods, this approach avoids genomic integration and reduces mutagenic risk, making it attractive for therapeutic use.

A common protocol combines epigenetic priming with lineage-directed differentiation. Initially, GCs are treated with 5-azacytidine (5-azaC), which inhibits DNMT activity and induces global DNA hypomethylation, reactivating pluripotency genes (*OCT4*, *SOX2*, *NANOG*) [17,55,60]. Short-term, low-dose exposure (24–72 h) is optimal for destabilizing the differentiated state, whereas prolonged or high-dose treatment may cause senescence or apoptosis, necessitating careful titration.

After priming, GCs are cultured in neural induction medium containing forskolin, bFGF, and retinoic acid (RA). Forskolin activates cAMP-dependent signaling and phosphorylates CREB, whereas RA functions as a morphogen that promotes neural specification. Together, these factors enhance neuroectodermal commitment and upregulate Nestin, GFAP, and βIII-tubulin (TUBB3) [61,62].

Biophysical cues also play a major role. Three-dimensional or low-attachment cultures promote sphere formation, enhance cell–cell interactions, and mimic the ovarian niche. Such environments stabilize stemness, reduce spontaneous differentiation, and activate integrin-dependent signaling controlling cytoskeletal reorganization [63,64,65].

Hydrogel scaffolds, alginate encapsulation, and Matrigel matrices further improve GC survival, proliferation, and lineage differentiation, demonstrating that matrix elasticity and composition can guide reprogramming outcomes [66].

Overall, combining epigenetic modulation, morphogen exposure, and engineered 3D microenvironments provides a robust platform for generating functional, patient-specific GC-derived cells. These protocols open new possibilities for modeling neurodegenerative and reproductive disorders and for personalized tissue engineering.

### 4.3. Advantages and Limitations

Epigenetic reprogramming of somatic cells into induced pluripotent-like states offers a transgene-free and non-integrative alternative to viral methods. Because no exogenous DNA is inserted, the approach avoids insertional mutagenesis and transgene reactivation, improving biosafety and clinical acceptability [67].

Granulosa cells are particularly responsive to such modulation. Their cyclic hormonal stimulation and transitional role in folliculogenesis maintain epigenetic flexibility, making them more susceptible to partial or lineage-specific reprogramming than many somatic cell types [68].

Additional technical advantages include

- Low cost and procedural simplicity of small-molecule use.
- Scalability for preclinical applications.
- Compatibility with 3D culture systems, enhancing differentiation outcomes.

However, several limitations persist (see Table 1):

1. Reprogramming efficiency remains variable and typically low, depending on donor origin, passage number, and hormonal context.
2. Residual epigenetic memory may bias lineage fate, limiting full reprogramming and long-term stability [69].
3. Incomplete chromatin remodeling may restrict activation of lineage-specific programs.
4. Functional validation in vivo is still lacking: the ability of reprogrammed GCs to integrate, survive, and function after transplantation must be tested in preclinical models [70].

In conclusion, chemically induced reprogramming of GCs represents a safe, cost-effective, and scalable approach for regenerative medicine. Yet, standardization, optimization, and in vivo verification remain essential before clinical implementation.

## 5. Comparative Perspective: GCs vs. oPSCs

Granulosa cells (GCs) and ovarian putative stem cells (oPSCs) represent two promising yet biologically distinct ovarian cell populations with potential applications in regenerative medicine, disease modeling, and tissue engineering. Despite sharing certain stem-like properties under experimental conditions, they differ in origin, accessibility, molecular identity, and developmental potential [1,71].

GCs are terminally differentiated somatic cells originating from the follicular microenvironment. They are routinely obtained during assisted reproductive procedures such as in vitro fertilization (IVF), intracytoplasmic sperm injection (ICSI), or somatic cell nuclear transfer (SCNT). They can also be recovered postmortem from slaughterhouse-derived ovaries. This makes them readily available and ethically acceptable cell sources for regenerative and reconstructive studies. Their molecular and epigenetic plasticity is induced primarily in vitro by exposure to 5-azacytidine (5-azaC), forskolin, or cytokines, which partially reprogram them toward pluripotency-associated and lineage-specific phenotypes [72].

In contrast, oPSCs—including very small embryonic-like stem cells (VSELs) and oogonial stem cells (OSCs)—reside in the ovarian cortex and exhibit intrinsic multipotency. When precisely isolated via immunomagnetic or flow cytometry-based methods, they show robust expression of pluripotency markers (OCT4, NANOG) and germline determinants (DDX4/VASA). Under defined conditions, oPSCs can differentiate into neurons, endothelial cells, osteoblasts, and even oocyte-like structures. However, their rarity, small size, and controversial identification limit their reproducibility and translational potential [73].

In summary, GCs and oPSCs constitute complementary cellular platforms. GCs are abundant, clinically accessible, and suitable for autologous applications, whereas oPSCs are rare but possess broader multipotency. Most supporting data derive from in vitro research; in vivo validation of functional integration remains limited. These differences define their distinct yet convergent roles in neuroregeneration, vascular engineering, and reproductive biology, as summarized in Table 2.

## 6. Applications in Regenerative Medicine

### 6.1. Neurological Applications

Ovarian granulosa cells (GCs) and ovarian putative stem cells (oPSCs) have emerged as promising cellular sources for regenerative medicine due to their plasticity, non-controversial origin, and capacity for lineage-specific differentiation. Although their developmental status and reprogramming mechanisms remain under investigation, both cell types have demonstrated potential in not only neural tissue engineering, but also vascular, and reproductive tissue engineering [30,74,75].

Under defined neuroinductive conditions involving retinoic acid, forskolin, bFGF, and low-adhesion systems, GCs and oPSCs can acquire neural-like phenotypes. These reprogrammed cells display neuron- and glia-like morphology and express neuroectodermal markers including βIII-tubulin (TUBB3), MAP2, NeuN, and GFAP [76].

Electrophysiological and co-culture studies with rat hippocampal neurons have confirmed delayed rectifier potassium currents, synchronized membrane fluctuations, and synaptic protein expression (synapsin-1, PSD-95), confirming their functional neuronal potential [77].

These findings highlight three major applications:

1. Modeling neurodegenerative diseases such as Alzheimer’s and Parkinson’s;
2. Drug screening and neurotoxicity testing using patient-derived lines;
3. Autologous neural cell therapy—especially in women—providing sex-matched compatibility [78].

The generation of neural cells from ovarian-derived sources also offers a unique tool for studying sex-specific aspects of brain aging, cognition, and neuroendocrine regulation [79].

### 6.2. Vascular Tissue Engineering

Ovarian PSCs, particularly those derived from porcine and human ovaries, exhibit robust endothelial differentiation when exposed to VEGF and cultured in Matrigel or vascular scaffolds. They express endothelial markers (VEGFR2, VE-cadherin, vWF, CD31), form capillary-like networks, and support neovascularization in vitro [23].

These properties enable potential applications in:

- Therapeutic revascularization and angiogenesis in ischemic diseases (e.g., peripheral artery disease, myocardial infarction);
- Pre-vascularization of engineered grafts, improving perfusion, survival, and tissue integration post-implantation;
- Restoration of microvascular networks damaged by chemo- or radiotherapy, particularly:
  - In the ovary, supporting oncofertility restoration after gynecological cancer treatment;
  - In the CNS, aiding vascular repair and potentially alleviating neurodegenerative or tumor-related pathologies.

Collectively, oPSCs and their endothelial derivatives represent versatile tools for promoting de novo angiogenesis and as pro-angiogenic components of multicellular constructs, strengthening the regenerative and rejuvenation potential of engineered tissues.

### 6.3. Reproductive Tissue Engineering

Given their ovarian origin, GCs and oPSCs are highly suitable for reproductive regenerative medicine, offering applications ranging from fertility preservation to endocrine restoration [80]. Key directions include:

- In vitro folliculogenesis: Both cell types can support follicle formation and maturation, enabling fertility preservation in cancer survivors [81];
- Three-dimensional-printed ovarian scaffolds: GCs embedded in biocompatible hydrogels restore partial endocrine function and support oocyte maturation in animal models [82].
- Bioartificial ovarian constructs: Integration of oPSCs enhances vascularization, organization, and graft longevity, providing a potential replacement for damaged ovarian tissue [83].
- Endocrine restoration: Autologous transplantation of steroidogenic GCs or oPSC-derived cells could serve as a physiological alternative to hormone replacement therapy, especially in women with premature ovarian insufficiency (POI) or polycystic ovary syndrome (PCOS) [84].

**Figure 4:**
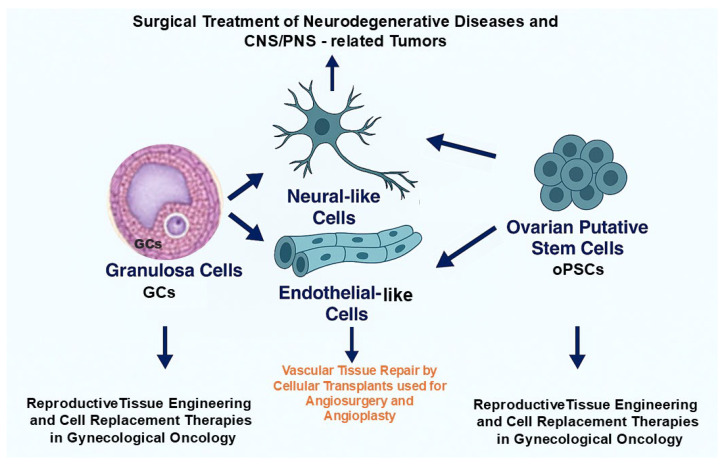
Potential implementation of therapeutic modalities based on grafting the ovarian-derived cells to personalized applications in regenerative and reconstructive medicine. Granulosa cells (GCs) and ovarian putative stem cells (oPSCs) may be exploited for neurological repair, vascular tissue engineering, and reproductive therapies, providing an ethically acceptable, autologous, and multipotent cell source.

Together, ovarian-derived cells such as GCs and oPSCs combine low immunogenicity with regenerative and anti-senescence properties, making them powerful candidates for personalized regenerative medicine in neurology, vascular tissue repair, and the restoration of female reproductive health (Figure 4).

## 7. Recent Challenges and Future Targets

Despite major advances in understanding the plasticity and regenerative potential of ovarian granulosa cells (GCs) and ovarian putative stem cells (oPSCs), several technical, biological, and regulatory barriers continue to limit their clinical translation. These challenges span the entire experimental pipeline, from isolation and reprogramming to functional validation and therapeutic application. Further progress will depend on standardizing isolation protocols, performing precise lineage tracing, implementing GMP-compliant reprogramming of GCs, and conducting comprehensive in vivo testing to confirm safety and efficacy.

### 7.1. Standardization of Isolation and Differentiation Protocols

The lack of reproducible and standardized procedures remains a major limitation. For oPSCs, inconsistencies in isolation techniques (e.g., magnetic vs. flow cytometry) and variability in surface marker definitions (SSEA-4, DDX4, CD133) hinder the comparison of results across studies and species.

For GCs, donor-related factors, such as age, hormonal milieu, follicular stage, and species differences, significantly influence reprogramming efficiency and the acquisition of stem-like characteristics.

Similarly, differentiation protocols targeting neuronal or endothelial lineages differ in medium composition, scaffold type, and morphogen concentrations (RA, forskolin, bFGF, VEGF), leading to inconsistent results and poor inter-laboratory reproducibility [85]. Developing standardized and validated differentiation systems will be essential for advancing translational research.

### 7.2. Genomic Stability and Epigenetic Memory

One of the most critical challenges in the reprogramming of GCs and oPSCs is the incomplete erasure of somatic epigenetic memory. Residual chromatin marks and DNA methylation patterns can bias lineage specification and reduce differentiation fidelity.

Moreover, prolonged exposure to demethylating agents such as 5-azacytidine (5-azaC) or 5-aza-2′-deoxycytidine (5-aza-dC), or extensive cell passaging, may result in chromosomal instability, telomere attrition, and mutagenic events, all of which threaten the genomic integrity of derived cells [86].

Addressing these issues through multi-omics profiling and long-term safety assessment will be crucial for ensuring therapeutic reliability.

### 7.3. Functional Validation In Vivo

While numerous in vitro studies have confirmed the differentiation of GCs and oPSCs into neural, endothelial, or follicle-like cells, functional integration in vivo remains largely unverified.

Only a few reports demonstrate that GC- or oPSC-derived neurons can integrate into host networks, form synapses, or restore function in models of neurodegeneration [30,87]. Similarly, endothelial or follicle-like constructs derived from oPSCs have rarely been tested for vascular patency, hormonal activity, or fertility restoration [88].

To advance toward clinical application, long-term in vivo assays assessing cell survival, maturation, immune tolerance, and functional contribution are urgently required.

### 7.4. Immunological and Ethical Considerations

Although both GCs and oPSCs can be derived autologously, the immunogenicity of these cells following reprogramming or prolonged culture remains unclear. GCs, often discarded during IVF or ICSI, represent an ethically acceptable cell source. In contrast, oPSCs—particularly oogonial stem cells (OSCs)—originate from primordial germline regions, raising bioethical and regulatory concerns, especially when considered for non-reproductive medical applications.

Furthermore, the existence and functional identity of OSCs and VSELs remain debated, complicating regulatory approval and public acceptance [89].

### 7.5. Regulatory Framework and Clinical Translation

Translating laboratory findings into clinically applicable therapies will require a robust regulatory framework and GMP-grade production pipeline.

- Reprogrammed GCs and oPSCs must meet rigorous safety, genomic integrity, and non-tumorigenicity standards.
- MA and FDA regulations currently lack clear classification criteria for stem cell-like products derived from reproductive tissues, complicating approval pathways.
- To date, no GC- or oPSC-based therapy has advanced beyond early preclinical studies; this statement should be verified prior to publication.
- Successful translation will depend on close collaboration among molecular biologists, clinicians, toxicologists, and regulatory authorities to ensure both efficacy and ethical compliance [90].

In summary, overcoming these challenges through standardized methodologies, multi-level validation, and ethical governance will be essential for unlocking the full regenerative potential of ovarian-derived cells in the coming decade.

## 8. Applicability of GCs and oPSCs to Somatic Cell Cloning-Based Assisted Reproductive Technologies in Mammals

The origin of nuclear donor cells plays a decisive role in determining the epigenetic reprogrammability of genomic DNA and the developmental efficiency of cloned embryos, fetuses, and offspring produced by somatic cell nuclear transfer (SCNT). This dependence has been demonstrated across numerous mammalian species, including livestock, laboratory, companion, and wild animals [91,92]. To date, only a limited range of donor cell types has been extensively characterized for their competence to support the full-term development of SCNT-derived conceptuses and progeny [93,94].

Within this context, ovarian-derived cell populations, including mural and cumulus granulosa cells (GCs) and female germline stem cells (FGSCs) or oogonial stem cells (OSCs), appear to offer notable developmental advantages over conventional donor cells such as fibroblasts or epithelial cells. Their application has been tested in

- Livestock species (e.g., cattle) [95];
- Laboratory animals (e.g., mice) [96];
- Other domesticated or wild mammals (e.g., dromedary camels, cynomolgus macaques) [97,98].

Comparative and mechanistic studies examining the epigenomic susceptibility of nuclear genomes derived from GCs and oPSCs to reprogramming during SCNT remain crucial for improving the overall success of cloning-based reproductive biotechnologies [99]. Such investigations could yield valuable insights into:

1. Enhancement of genetic merit and quantitative trait locus (QTL) expression in cloned livestock [100];
2. Transgenic and translational research supporting applications in biopharmacology, regenerative medicine, and reconstructive surgery [101,102];
3. Genetic rescue programs aimed at endangered species and rare livestock breeds [103];
4. Fundamental studies exploring the cellular and molecular determinants of epigenetic reprogramming that govern the developmental competence of cloned embryos reconstructed with somatic cell nuclei [99].

In summary, ovarian-derived cells, such as GCs and oPSCs, represent a promising, biologically privileged source of nuclear donors for SCNT, combining high epigenetic responsiveness with ethical acceptability and broad applicability in both agricultural and biomedical cloning contexts.

## 9. Comprehensive Summary and Paramount Conclusions

Mammalian granulosa cells (GCs) and ovarian putative stem cells (oPSCs) are increasingly recognized as accessible, versatile, and ethically favorable candidates for regenerative medicine. Once considered solely as components of ovarian physiology, supporting oocyte growth and steroid hormone synthesis, these cells are now viewed as dynamic somatic populations with remarkable plasticity and reprogramming potential.

Granulosa cells, easily obtained during gynecological procedures or assisted reproductive technologies (IVF, ICSI, SCNT), demonstrate significant epigenetic flexibility. Through chemical, non-integrative reprogramming, they can express pluripotency markers and differentiate into neural- or endothelial-like cells, avoiding the genomic risks associated with viral vectors. This transgene-free approach enhances both the biosafety and translational potential of GCs.

Ovarian PSCs, including oogonial stem cells (OSCs) and very small embryonic-like stem cells (VSELs), isolated from the ovarian cortex, express pluripotency and mesenchymal markers and can differentiate into neuronal, endothelial, adipogenic, and osteogenic lineages. Their multipotency and responsiveness to niche cues make them powerful tools for vascular, neural, and reproductive tissue engineering.

Despite these promising features, several obstacles still limit clinical translation:

- Protocol variability in isolation and differentiation impedes reproducibility;
- Incomplete epigenetic reprogramming and potential genomic instability raise safety concerns;
- Functional in vivo validation of graft survival and integration remains scarce;
- Ethical and regulatory frameworks for reproductive tissue-derived cells are still evolving.

Nevertheless, the autologous origin, non-genomic reprogramming, and defined differentiation capacity of GCs and oPSCs support their future use in personalized and translational regenerative therapies. Integration of single-cell transcriptomics, epigenomic mapping, and cross-species comparative studies may help overcome current limitations and strengthen their biomedical relevance.

Among ovarian-derived cells, GCs currently offer the most practical and stable source for regenerative applications. They can be obtained postmortem or during ART procedures, exhibit low immunogenicity, and maintain karyotypic stability, making them suitable for autologous grafting in female patients with ovarian dysfunction, such as primary ovarian insufficiency (POI), polycystic ovary syndrome (PCOS), or benign or malignant ovarian neoplasms. Conversely, oPSCs, though requiring further validation for safety and reproducibility, may serve as complementary cell platforms alongside GCs, enabling deeper exploration of cell fate plasticity and lineage commitment in preclinical models, and development of cell-based therapies applicable to reproductive, vascular, and neuroregenerative medicine.

Moreover, preliminary findings indicate that early oocyte-associated somatic cells (mural and cumulus GCs) or female germline cells (OSCs) display superior epigenetic reprogrammability as nuclear donors in SCNT-based cloning across various mammalian species. However, systematic, species-specific, and cell-type-controlled comparative studies remain essential to confirm these advantages and optimize their biotechnological application.

In conclusion, ovarian-derived cell populations—granulosa cells and ovarian putative stem cells—constitute a versatile, safe, and biologically meaningful foundation for regenerative medicine. Their full translational potential will depend on continued advances in bioengineering, single-cell analytics, and ethical oversight, as well as collaborative, cross-disciplinary research that bridges reproductive biology and clinical regenerative technologies. With these developments, ovarian-derived cells may soon transition from experimental systems to clinically relevant therapeutic platforms.

## 10. Concluding Remarks and Future Perspectives

Ovarian-derived cells, particularly granulosa cells (GCs) and ovarian putative stem cells (oPSCs), represent a promising and ethically acceptable cellular platform for regenerative medicine. Their unique combination of accessibility, reprogrammability, and lineage plasticity distinguishes them from other somatic or pluripotent cell sources. Granulosa cells, obtainable during standard reproductive procedures, show high epigenetic responsiveness and can be chemically reprogrammed without genetic modification, supporting their potential use in autologous neuro, vascular, and ovarian tissue reconstruction. In parallel, oPSCs, including oogonial stem cells (OSCs) and very small embryonic-like stem cells (VSELs), exhibit intrinsic multipotency and can differentiate into derivatives of all three germ layers under controlled conditions.

Despite these advances, several challenges remain, including protocol standardization, genomic stability, in vivo validation, and the need for coherent regulatory guidelines. Addressing these gaps through integrated molecular, epigenetic, and functional analyses will be essential to translate current experimental achievements into clinical applications.

To sum up, GCs and oPSCs bridge reproductive biology and regenerative medicine, providing a biologically relevant, patient-specific, and ethically sustainable foundation for future cell-based therapies. With continued progress in bioengineering, single-cell analytics, and translational collaboration, ovarian-derived cells may soon progress from laboratory exploration to clinically viable therapeutic strategies for restoring tissue integrity and function in reproductive and systemic disorders.

## Figures and Tables

**Table 1:** Advantages and limitations of chemically triggering the epigenetic reprogramming of granulosa cells.

Advantages	Limitations
**Non-integrative method**: Avoids genome modification, reducing oncogenic risk.	**Low and variable efficiency**: Responses to chemical agents depend on cell context and the protocol used.
**Transgene-free**: Does not require viral vectors or exogenous gene delivery.	**Epigenetic memory**: Residual somatic signature may bias differentiation outcomes.
**Safer for clinical translation**: Reduced mutagenic potential.	**Incomplete reprogramming**: Limited chromatin remodeling may impair full lineage commitment.
**Cost-effective and scalable**: Small molecules are easy to produce and apply.	**Limited in vivo validation**: Functional integration and long-term behavior are still unproven.
**GCs are highly responsive**: Naturally plastic due to the hormonal environment.	**Batch-to-batch variability**: Different donor sources or ovarian stages may impact reproducibility.
**Compatible with 3D and niche-mimicking systems.**	**Need for prolonged culture or complex combinations of agents.**

**Table 2:** Comparative characterization of parameters related to provenance, functional attributes, molecular determinants, and applicability of granulosa cells (GCs) and ovarian putative stem cells (oPSCs).

Parameter	Granulosa Cells (GCs)	Ovarian Putative Stem Cells (oPSCs)
**Origin**	Follicular granulosa layer (coelomic epithelium-derived).	Ovarian cortex (tunica albuginea and stromal niche).
**Accessibility**	High (e.g., denudation of oocytes from cumulus and mural granulosa cells during IVF, ICSI, and SCNT-based cloning procedures; postmortem retrieval from ovarian follicle-derived aspirates).	Low (require FACS/MACS; rare population).
**Marker expression**	Inducible expression of OCT4, SOX2, and NANOG under in vitro reprogramming.	Endogenous expressions of OCT4, NANOG, VASA, DDX4, CD90, and CD105.
**Plasticity** **mechanism**	Requires chemically assisted reprogramming (5-azaC, RA, LIF, and bFGF).	Innate multipotency; spontaneous or induced differentiation.
**Differentiation** **capacity**	Neural, glial, and endothelial (after reprogramming).	Ectodermal (neurogenic), endodermal (endotheliogenic), mesodermal (adipogenic, osteogenic), and possibly gametogenic (germline-specific).
**Stability**	Genetically stable; matched to donor genome.	Stability under debate; heterogeneous population suspected.
**Immunogenicity**	Low (autologous source).	Unknown; more studies needed.
**Clinical potential (possible** **applicability)**	Toxicology; disease modeling; regenerative medicine strategies based on undifferentiated GC-mediated repair of ovarian tissues or focused on the potential usefulness of transdifferentiated GC culture engineering (e.g., therapeutic approaches either with the aid of auto-/allo-/xenogenetically grafted GC-descended neuronal and glial cells suitable for surgical treatment of neurodegenerative diseases or with the aid of GC-derived endothelial cell transplants relevant to angiology, angiosurgery, and angioplasty).	Regenerative therapies based on auto-/allo-/xenogenetically grafting either oPSCs or their transdifferentiated cell derivatives, potentially applicable to tissue repair of: (1) female gonads and other compartments of reproductive system, or (2) target cardiac/pulmonary/cerebral/peripheral vascular bed, or (3) central nervous system (CNS) and peripheral nervous system (PNS); reconstructive modalities of cell/tissue engineering potentially applicable to gynecological oncology and preservation of fertility or oncofertility; all the above-indicated therapies/modalities remain largely preclinical and have not yet advanced to clinical trials.

## Data Availability

Not applicable.

## Abbreviations

5-azaC	5-Azacytidine; DNA methyltransferase inhibitor
5-aza-dC	5-Aza-2′-deoxycytidine (decitabine); DNA methyltransferase inhibitor
AKT	Serine/threonine protein kinase involved in cell survival signaling
ALB	Albumin
AMPAR	Ionotropic glutamate receptor mediating fast excitatory transmission
ARTs	Assisted reproductive technologies
ATAC-seq	Assay for transposase-accessible chromatin with sequencing
bFGF	Basic fibroblast growth factor
CACNA1C	Gene encoding L-type voltage-gated calcium channel subunit α1C
CD	Cluster of differentiation
c-KIT	Proto-oncogene encoding receptor tyrosine kinase CD117
CpG	DNA region rich in cytosine–guanine dinucleotides
CREB	cAMP response element-binding protein (transcription factor)
CYP3A4	Cytochrome P450 enzyme involved in drug metabolism
c-MYC	Proto-oncogene encoding transcription factor regulating proliferation
CNS	Central nervous system
DAZL	Deleted in azoospermia-like; germ cell RNA-binding protein
DDX4 (VASA)	DEAD-box RNA helicase; germline marker
DNMT	DNA methyltransferase
DNMTi	DNA methyltransferase inhibitor
DPPA3 (STELLA)	Maternal factor regulating DNA methylation in germ cells
ECM	Extracellular matrix
EPCs	Endothelial progenitor cells
ESCs	Embryonic stem cells
FABP4	Fatty acid-binding protein 4
FACS	Fluorescence-activated cell sorting
FGSCs (PGCs)	Female germline stem cells (primordial germ cells)
Fluo-4 AM	Calcium-sensitive fluorescent indicator dye
GCs	Granulosa cells
GFAP	Glial fibrillary acidic protein
HAT	Histone acetyltransferase
HDAC	Histone deacetylase
HGF	Hepatocyte growth factor
HRT	Hormone replacement therapy
ICSI	Intracytoplasmic sperm injection
IFITM3 (FRAGILIS)	Interferon-induced transmembrane protein 3; germline marker
iPSCs	Induced pluripotent stem cells
IVF	In vitro fertilization
KCNQ2	Voltage-gated potassium channel subunit regulating excitability
KLF4	Krüppel-like factor 4; zinc finger transcription factor
LIF	Leukemia inhibitory factor
MACS	Magnetic-activated cell sorting
MAP2	Microtubule-associated protein 2
MAPK/ERK	Mitogen-activated protein kinase/extracellular signal-regulated kinase
MSCs	Mesenchymal stem cells
NANOG	Homeobox transcription factor maintaining pluripotency
NeuN	Neuronal nuclei antigen; marker of mature neurons
NMDAR	N-methyl-D-aspartate receptor; ionotropic glutamate receptor
NPY	Neuropeptide Y
NR1	NMDA receptor subunit 1
OCT4 (POU5F1)	Octamer-binding transcription factor 4; pluripotency regulator
oPSCs	Ovarian putative stem cells
OSCs	Oogonial stem cells
PECAM-1	Platelet–endothelial cell adhesion molecule-1 (CD31)
PI3K	Phosphatidylinositol 3-kinase
PNS	Peripheral nervous system
PCOS	Polycystic ovary syndrome
POI/POF	Primary ovarian insufficiency/premature ovarian failure
PPARγ	Peroxisome proliferator-activated receptor gamma
PSD-95	Postsynaptic density protein 95
qRT-PCR	Quantitative real-time polymerase chain reaction
QTLs	Quantitative trait loci
RA	Retinoic acid
RUNX2	Runt-related transcription factor 2
SCNT	Somatic cell nuclear transfer
SCN1A	Sodium voltage-gated channel α subunit 1
SOX2	SRY-box transcription factor 2
SOX9	SRY-box transcription factor 9
SSEA-4	Stage-specific embryonic antigen 4
STAT3	Signal transducer and activator of transcription 3
TRA-1-81	Surface antigen of pluripotent stem cells
TUBB3	Class III β-tubulin
VSELs	Very small embryonic-like stem cells
VEGF	Vascular endothelial growth factor
VEGFR2	Vascular endothelial growth factor receptor 2
VEGFR3	Vascular endothelial growth factor receptor 3
VGCCs	Voltage-gated calcium channels
vWF	Von Willebrand factor
Wnt	Family of signaling proteins regulating cell fate and development
